# Peat-derived hard carbon electrodes with superior capacity for sodium-ion batteries†

**DOI:** 10.1039/d0ra03212c

**Published:** 2020-05-27

**Authors:** Anu Adamson, Ronald Väli, Maarja Paalo, Jaan Aruväli, Miriam Koppel, Rasmus Palm, Eneli Härk, Jaak Nerut, Tavo Romann, Enn Lust, Alar Jänes

**Affiliations:** Institute of Chemistry, University of Tartu Ravila 14a 50411 Tartu Estonia alar.janes@ut.ee; Institute of Ecology and Earth Sciences, University of Tartu Ravila 14a 50411 Tartu Estonia; Institute Electrochemical Energy Storage (EM-IEES), Helmholtz-Zentrum für Materialien und Energie GmbH Hahn-Meitner-Platz 1 14109 Berlin Germany

## Abstract

Herein we demonstrate how peat, abundant and cheap biomass, can be successfully used as a precursor to synthesize peat-derived hard carbons (PDCs), applicable as electrode materials for sodium-ion batteries (SIB). The PDCs were obtained by pre-pyrolysing peat at 300–800 °C, removing impurities with base–acid solution treatment and thereafter post-pyrolysing the materials at temperatures (*T*) from 1000 to 1500 °C. By modification of pre- and post-pyrolysis temperatures we obtained hard carbons with low surface areas, optimal carbonization degree and high electrochemical Na^+^ storage capacity in SIB half-cells. The best results were obtained when pre-pyrolysing peat at 450 °C, washing out the impurities with KOH and HCl solutions and then post-pyrolysing the obtained carbon-rich material at 1400 °C. All hard carbons were electrochemically characterized in half-cells (*vs.* Na/Na^+^) and capacities as high as 350 mA h g^−1^ at 1.5 V and 250 mA h g^−1^ in the plateau region (*E* < 0.2 V) were achieved at charging current density of 25 mA g^−1^ with an initial coulombic efficiency of 80%.

## Abstract 2

A synthesis method has been developed to turn peat, cheap biomass into hard carbons that demonstrate high capacity and excellent sodium storage capability as anode material in sodium-ion batteries.

## Graphical abstract

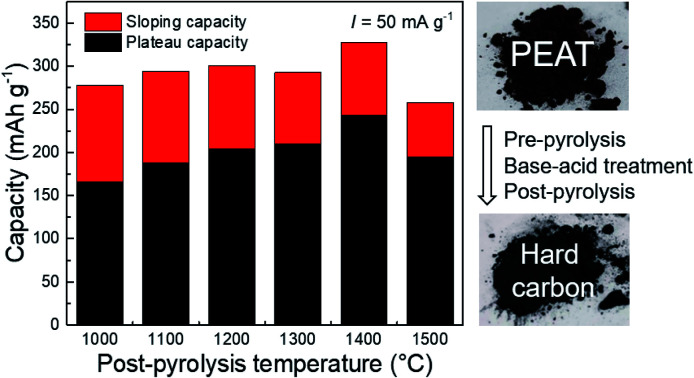

## Introduction

In recent years, there has been a remarkable rise in the significance of renewable energy resources.^1,2^ However, the energy produced by these resources (wind, solar *etc.*) also needs to be stored because of the intermittent nature of renewable energy sources. For example, lithium-ion batteries (LIBs) are already used to store photovoltaic electricity.^3^ Lithium-ion batteries have many distinctive advantages like high energy density, nominal voltage (4 V) and long cycle life.^4^ However, the growing cost and scarcity of lithium and other elements used in LIBs are an increasing concern.^5^ Sodium has similar electrochemical properties to lithium, but is larger and heavier, which lowers the theoretical energy density^8^ and the specific energy of sodium-ion batteries (SIBs). However, because of its low price and high abundance^6,7^ SIBs are promising in stationary energy storage applications – such as storing electricity from renewable energy sources.^2,8,9^

In addition, the absence of suitable negative electrode materials is limiting the commercialization of SIBs.^10^ Graphite, which has very high theoretical capacity in LIBs (372 mA h g^−1^),^11,12^ is not applicable in SIBs since the theoretical capacity of graphite in SIB is only 35 mA h g^−1.^^13^ Since Na^+^ is bigger than Li^+^, the intercalation of Na^+^ into graphite stretches the C–C bonds in graphite-like structures more than Li. Consequently, it has been shown, that the Na–graphite intercalation compound (Na–GIC) is highly unstable.^14^

Different types of materials have been explored as the negative electrodes for SIB, including metal oxides/alloys, but carbon materials (hard carbons in particular with specific properties) have emerged as the most promising of all the proposed materials.^15^ Hard carbons have demonstrated high capacities and structural properties that make them potentially applicable for the use as negative electrodes in SIBs.^16^

Hard carbons have attracted attention due to their high plateau capacity at low potential (*E* < 0.2 V Na/Na^+^) which enables high specific energy.^17^ There are many specific structural properties of hard carbon that influence the electrochemical performance of SIBs. It has been shown that some carbons with low specific surface area and a specific degree of graphitization are preferable for use in SIBs.^18,19^ Thus, choosing the hard carbon with optimal properties has a great impact on the performance of the SIB, especially on the irreversible capacity (IRC) of the 1^st^ cycle and initial charging/discharging coulombic efficiency (ICE). This is related to the formation of the solid electrolyte interface (SEI) layer on the surface of a carbon electrode. SEI forms during the 1^st^ charging cycle when sodium ions intercalate into the hard carbon and the non-aqueous electrolyte partly decomposes.^20^ Many of the intercalated sodium ions react with the decomposition products of the electrolyte and are therefore irreversibly lost to the formation of SEI layer.^18,21^ Therefore, the lower surface area of carbon is preferable since it also means less surface area for the formation of SEI.

Hard carbons can be synthesized from many different precursor materials, such as polymers, sugars^22,23^ or biomass.^16^ Some biomass-derived hard carbons have been shown to be highly effective as SIB negative electrode materials. For example, the capacities of 181, ∼280, 350 and 360 mA h g^−1^ were achieved for pomelo peels,^24^ coconut oil,^25^ lotus stem^26^ and carbonized leaves^27^ based SIBs, respectively.

In this study, highly decomposed Estonian peat was used as a precursor for the synthesis of hard carbon. The natural cellular structure of peat may be useful in order to obtain the hard carbon structure necessary to promote sodium ion (de)intercalation. The peat moss derived carbon previously used in SIB application has demonstrated capacities up to 275 mA h g^−1^, with a plateau of 180 mA h g^−1^ below 0.2 V *vs.* Na/Na^+^.^28^ One of the main advantages of peat is that it can be excavated from bogs and collected effectively in millions of tons, in comparison with the tediousness of collecting, say, banana peels from recycled waste.

Sometimes a serious drawback of biomass precursors is their impurity content. Some research on the removal of impurities from the biomass precursors has been conducted,^29–31^ but universal solutions are not yet available. The most pronounced problem with the inorganic impurities is their inactiveness in electrochemical processes by lowering the specific energy of the material by acting as deadweight. Although a great extent of research has been made to establish the effect of the pyrolysis temperature on the performance of biomass-derived hard carbons in SIBs,^29,30,32,33^ fewer studies have been conducted to analyze the effect of pyrolysis temperature in a multi-step carbon synthesis process on Na^+^ storage characteristics. In this study, a multi-step synthesis process has been developed, where the peat biomass precursor is pre-pyrolysed at a lower temperature (<1000 °C), thereafter washed with KOH and HCl aqueous solutions and finally post-pyrolysed at a higher temperature (>1000 °C). Both the pre- and post-pyrolysis temperatures have been varied systematically and, also, the effect on structural properties of the cleaning (KOH and HCl treatment) of the carbon material in between the pyrolysis stages has also been studied. The goal was to examine the effect of the adjustment and optimization of these synthesis steps on obtained peat derived hard carbons' properties. Furthermore, influence of pre- and post-treatment steps of obtained hard carbons on the energy storage capability of SIBs has been demonstrated.

## Experimental

### Synthesis of hard carbon materials

**Fig. 1:**
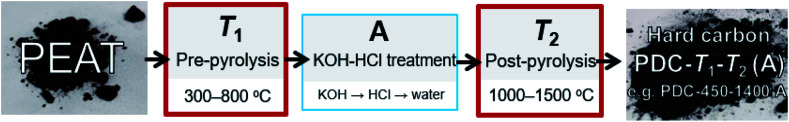
Synthesis steps and nomenclature of obtained hard carbons: *T*_1_ marks the pre-pyrolysis temperature (synthesis of peat to carbonaceous material). The letter A appended to the ending of hard carbon notation indicates that between two pyrolysis stages, the material was treated with KOH and HCl. *T*_2_ marks the post-pyrolysis temperature (synthesis of carbonaceous material to carbon).

Highly decomposed peat was obtained from Möllatsi bog in Tartumaa, Estonia. The peat-derived carbon (PDC) was prepared in three steps. Firstly, the peat was washed with water (MilliQ^+^), then homogenized and pre-pyrolysed for 3 h at fixed temperatures from 300 to 800 °C (at 100 °C interval). Obtained carbonaceous material was thereafter stirred for 2 h in 20% KOH aqueous solution at 70 °C. Thereafter, 50 vol% HCl solution was added until the pH of the solution reached ∼1, and the solution was stirred overnight. The treatment with HCl probably contributed to the increased content of chloride anions in the carbonaceous materials (Table S1 in ESI†). In order to thoroughly clean and eliminate the residual chlorine anions, the carbonaceous materials were stirred overnight in MilliQ^+^ water and dried in a vacuum oven. As a final crucial step, the carbonaceous materials were post-pyrolysed at fixed temperatures from 1000 to 1500 °C with a 100 °C interval. The pre-pyrolysis was carried out in a quartz-tube furnace (Carbolite) and post-pyrolysis in an Al_2_O_3_-tube furnace (Carbolite Gero). The nomenclature and synthesis of obtained hard carbons are shown in Fig. 1.

### Characterization of hard carbon materials

The structure of the synthesized hard carbons was investigated with X-ray diffraction (XRD), Raman spectroscopy, gas sorption (with N_2_ and Ar), scanning electron microscopy coupled with energy dispersive X-ray spectrometry (SEM-EDX), transmission electron microscopy (TEM) and laser powder diffraction (LPD) methods. The thermogravimetric analysis (TGA) was performed to evaluate the burn-off of peat's organic content during pre-pyrolysis. In order to evaluate the effect of the KOH–HCl treatment on hard carbon, the elemental composition was investigated before and after the treatment using X-ray fluorescence spectrometry (XRF).

The XRF measurements were performed using Rigaku wave-dispersive X-ray fluorescence spectrometer SZX Primus II. Rh primary radiation with a power of 3 kW was used. All elements from C to U were measured from 20 mm pellets into which the powder samples were pressed. The pellets were covered with a 6 μm thick polyester plastic (Mylar, DuPont), the signal of which was subtracted. The results were calculated with the ScanQuantX method. XRD measurements were executed with Bruker X-ray diffractometer D8 Advance. Ni-filtered Cu Kα radiation was used. Raman spectra were acquired with Renishaw inVia Raman microscope and Ar laser with a wavelength of 514 nm was used for excitation.

TEM micrographs have been selected from TEM measurement data conducted using JEOL JEM-2100 (JEOL GmbH, Eching, Germany) instrument with an acceleration voltage of 200 kV.

For each powder sample, multiple spectra were measured from different areas on the sample and the averaged information of 3–6 spectra was used for further analysis. The first-order Raman spectra were deconvoluted using the Gaussian and Lorentz distribution functions. Gas sorption measurements were carried out with TriFlex and ASAP 2020 systems. The samples were degassed for more than 3 h at 0.05 mbar. Nitrogen (Linde Gas, 6.0) and argon (Linde Gas, 6.0) sorption measurements were conducted at 77 K and 87 K, correspondingly.

The specific surface area of the hard carbon materials was calculated according to BET (N_2_) and NLDFT (Ar) theories. For some materials, pore size distributions were also calculated, using the SAIEUS carbon N2-77 2D-NLDFT heterogeneous surface model.^34^

Thermogravimetric analysis (TGA) of peat was performed with NETZSCH STA449F3 using an Al_2_O_3_ crucible. During the measurement, the temperature increased from 25 to 1000 °C with a heating up rate of 10 °C min^−1^. The flow rate of nitrogen (Linde Gas, 3.0) was 50 cm^3^ min^−1^. Laser powder diffraction (LPD) was used to measure the relative particle sizes of obtained pre-pyrolysed carbonaceous materials (PDC-*T*_1_). It was conducted with a Microtrac S3500 Bluewave instrument using ultrasound to separate agglomerates that may have formed.

### Electrochemical characterization of hard carbon materials

The obtained hard carbon was ground and mixed with conductive carbon black Super P (Alfa Aesar) and polyvinylidene difluoride (PVDF) in a 75 : 15 : 10 weight ratio in *N*-methyl-2-pyrrolidone (NMP, Sigma-Aldrich, 99.5%) mixture to form electrode slurry. The resulting mixture was cast onto aluminium foil using a doctor blade and thereafter dried on a hotplate. Electrode discs with a geometric area of 2 cm^2^ were cut and dried under vacuum at 120 °C for 16 h. The mass-loading of active material in most cases was around 1.75 mg cm^−2^.

Electrochemical characterization was carried out in the half-cells using sodium metal (Acros, 99.8%) as a counter electrode and 1.55 mm glass fibre as a separator EL-Cell GmbH in the 2032-type coin cells. Measurements were carried out comparing two electrolytes: 1 M NaClO_4_ (Alfa Aesar, 99%) solution in 1 : 1 (v/v) ethylene carbonate (EC, Sigma-Aldrich, 99%) and diethyl carbonate (DEC, Sigma-Aldrich, 99%) mixture and 1 M NaPF_6_ solution in 1 : 1 (v/v) EC and propylene carbonate (PC, Sigma-Aldrich, 99.7%). All cells were assembled in an Ar-filled glove box (O_2_ and H_2_O < 0.1 ppm).

Cycling performance of the PDC electrodes has been investigated using galvanostatic charge/discharge method at current densities ranging from 25 to 2000 mA g^−1^. The current density and capacity values have been expressed per active material of the working electrode, and the cycling has been carried out from 0.005 to 1.5 V (*vs.* Na/Na^+^).

## Results and discussion

### SEM-EDX and TEM measurements

**Fig. 2:**
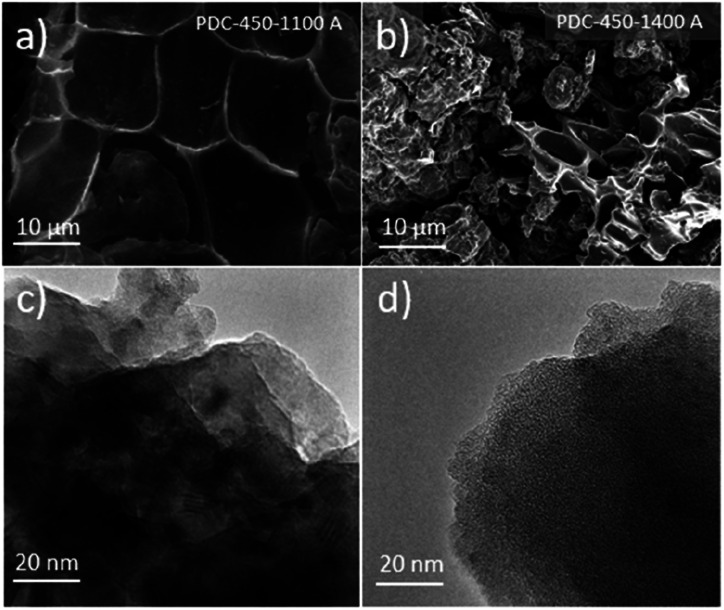
SEM images for PDC-450-1100 A (a) and PDC-450-1400 A (b). TEM images for PDC-500 (c) and (d) taken from different sites of the material.

SEM images demonstrate that peat's natural cellular structure is still preserved (Fig. 2a and b), even after the fairly destructive synthesis steps. SEM-EDX of selected carbon samples demonstrates that the increase in the post-pyrolysis temperature gives rise to hard carbons with higher carbon content and lower impurity content (Table S1†). However, the change in carbon content does not appear to have much influence on the morphology or topography of the carbon samples prepared (Fig. S1†).

TEM images taken of PDC-500 (Fig. 2c and d) indicate a mix of amorphous carbon and some (Ca(OH)_2_ and Fe(OH)_2_) crystals embedded within the carbon structure, as demonstrated by XRD and XRF measurements. However, because of the too amorphous structure and existence of some impurities, it was not possible to determine the layer spacing from the TEM images.

### Thermogravimetric analysis and laser powder diffraction

**Fig. 3:**
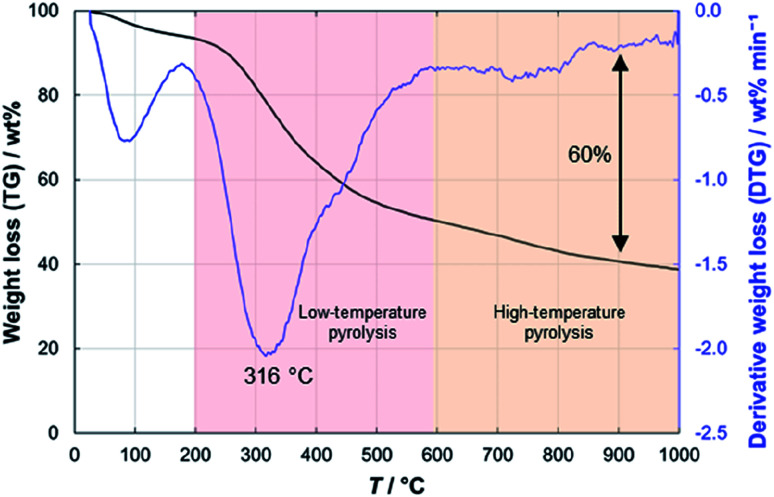
Thermogravimetric analysis results for peat.

In the thermogravimetric analysis of the precursor peat, several characteristic stages during the heating process can be distinguished (Fig. 3). Firstly, around 100 °C water evaporates from the sample and causes a 5–6 wt% decrease in the sample weight. At 200 °C, the low-temperature pyrolysis stage begins, which accounts for 45 wt% decrease in the mass of the sample. In the final, high-temperature stage of pyrolysis at >600 °C, the slower weight loss takes place. Finally, at 1000 °C about 40 wt% of the initial mass of peat remains. Nearly similar patterns have been observed for peats of different origin.^35–37^

The particle sizes of only pre-pyrolysed PDCs were also measured, using the laser diffraction method. At *T*_1_ = 600 °C, where the burn-off process reached a plateau, the formulated particles were significantly smaller, compared to the other only pre-pyrolysed PDCs. The particle sizes of all the measured PDCs were around 6 μm. However, for PDC-600, the number was almost twice as small (Table S2†).

### X-ray fluorescence spectroscopy

The XRF studies of the precursor peat show that the peat contains significant amounts of Ca and Fe (Table S2†) which would be electrochemically inactive and therefore needed to be removed. XRF data also shows that the materials pre-pyrolysed at lower temperatures contain more Ca and Fe (*T*_1_ = 450 °C, *w*_Ca%_ = 19.3) than materials pre-pyrolysed at higher temperatures (*T*_1_ = 800 °C, *w*_Ca%_ = 8.2). However, after KOH and HCl treatment steps the materials pre-pyrolysed at lower temperatures contained less Ca (and Fe) (*T*_1_ = 450 °C, *w*_Ca%_ = 0.38) than the materials pre-pyrolysed at higher temperatures (*T*_1_ = 800 °C, *w*_Ca%_ = 2.7).

Therefore, the biggest effect of KOH and HCl treatment steps was to reduce the Ca-content of pre-pyrolysed carbons, however, the content of Fe also decreased (Table S2†). This phenomenon can be explained by the encapsulation of Fe and Ca compounds during pre-pyrolysis at higher temperatures.

### X-ray diffraction analysis

**Fig. 4:**
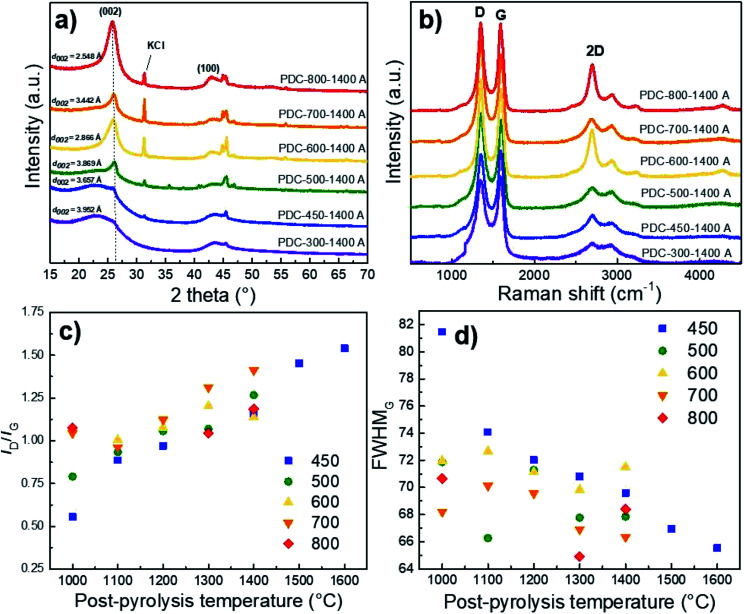
The effect of pre-pyrolysis temperature on the XRD diffractograms (a), Raman spectra (b), *I*_D_/*I*_G_ ratio (c), and FWHM_G_ calculated from Raman spectra of hard carbons with various post-pyrolysis temperatures (*T*_2_, *x*-axis) and with pre-pyrolysis temperatures (*T*_1_) ranging from 400 to 800 °C (noted in figure) (d).

XRD results show that Ca and Fe exist mostly in the form of oxides/hydroxides and that the KOH and HCl treatment steps cause a high KCl content in the materials if *T*_1_ > 500 °C has been applied (Fig. S2†). It must be said that higher pyrolysis temperature always contributes to increased graphitization.^38,39^ However, Fig. 4a demonstrates the high content of non-carbon compounds in PDC pre-pyrolysed at 600 °C and post-pyrolysed at 1400 °C (noted as PDC-600-1400 A) and a high *d*_002_ reflex, indicating its graphitization. Whereas the impurity content in PDC-700-1400 A is lower, as is the *d*_002_ reflex, being therefore less graphitized. The hard carbon synthesized at the highest pre-pyrolysis temperature (PDC-800-1400 A) is nevertheless the most graphitized—therefore both PDC-800-1400 A and PDC-600-1400 A demonstrate that the content of non-carbon compounds and temperature definitely play a role in the graphitization step. Similar phenomenon, where Fe acts as a catalyst for graphitization in biomass precursor, has been found in literature.^40^ It must be noted, that at *T*_1_ = 600 °C (as discussed earlier), significantly smaller particles were formed and it is intuitive that graphitization would occur more readily in the case of smaller particles because of extra space for atoms to reorganize. Materials with low impurity contents (*T*_1_ < 500 °C), also display small *d*_002_ peaks, indicating the formation of local graphitic domains, a characteristic of high capacity hard carbons.^19^

The average layer spacing was calculated for obtained hard carbons using DIFFRAC.EVA software. As a general trend, the interlayer spacing decreases with increasing the pre-pyrolysis temperature. However, PDC-600-1400 A is an exception, which is consistent with Raman spectroscopy and gas sorption measurements. The main reason is the formation of much smaller particles during the pre-pyrolysis at 600 °C, as discussed above. The calculated interlayer spacing values confirm that carbon is graphitized to a higher degree at pre-pyrolysis temperatures in the range of 600–800 °C. However, the interlayer spacing values calculated for PDC-(300-500)-1400 A are characteristic for hard carbons, which agrees well with Raman spectroscopy and gas sorption measurement results.

### Raman spectroscopy

The correlation between both graphitization (rise of sp^3^ carbon content) and non-carbon compound content is also supported by the shape of Raman spectra. The materials synthesized at *T*_1_ = 600 °C and *T*_1_ = 800 °C and post-pyrolysed at *T*_2_ = 1400 °C demonstrate a distinctive 2D peak, indicating the existence of graphene layers and turbostratic carbon structure on the surface.^41^ The carbon materials with more distinct second-order region in their Raman spectra (*T*_1_ > 600 °C) also demonstrate *d*_002_ reflexes with higher intensity in the XRD measurements, indicating a higher degree of graphitization (Fig. 4b).

Compared to the full width at half maximum values of the D-peaks (FWHM_D_), the G-peaks (FWHM_G_) remain more or less the same with the rise of post-pyrolysis temperature. This indicates that the burn-off takes place at the expense of amorphous rather than graphitic carbon, which contributes to graphitization at higher pyrolysis temperatures. This is further supported by Fig. 4c and d, which demonstrate that the *I*_D_/*I*_G_ ratio increases and FWHM_G_ decreases with the post-pyrolysis temperature, indicating somewhat higher disordering in the carbon structure, similar phenomena and tendencies have been reported in literature.^42,43^

### Gas sorption results

**Fig. 5:**
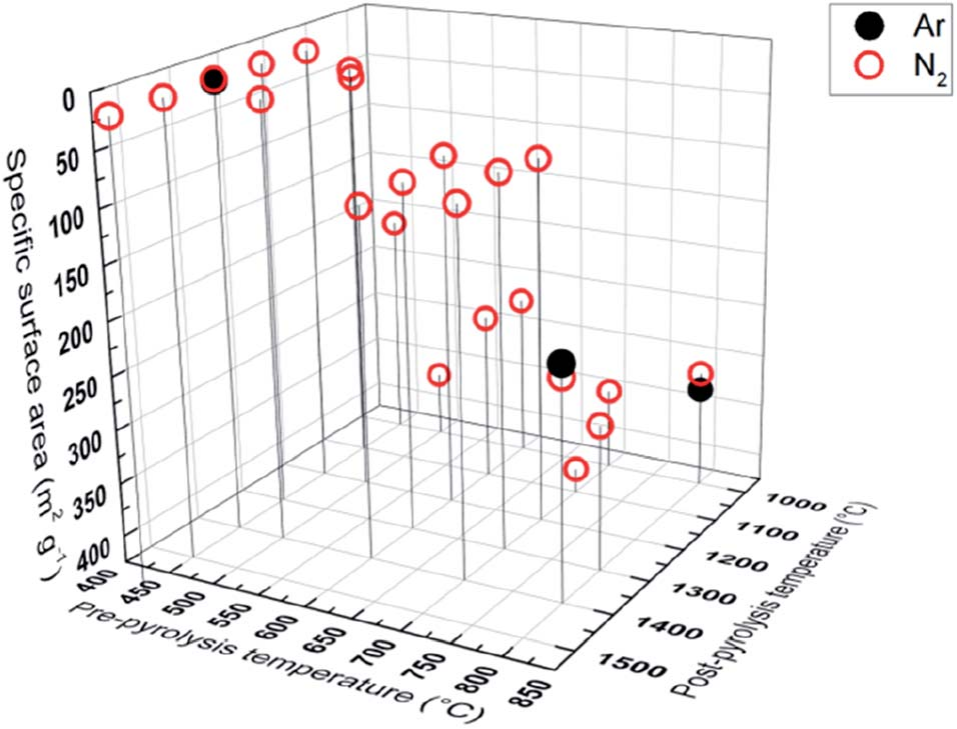
Gas sorption measurements with Ar and N_2_, showing correlation between pyrolysis temperature and specific surface.

Specific surface area, calculated from gas (N_2_, Ar) sorption data, increases with pre-pyrolysis temperature *S*_BET_ = 6 m^2^ g^−1^ for PDC-450-1400 A, while *S*_BET_ = 222 m^2^ g^−1^ for PDC-800-1400 A (Fig. 5). Compared to the pre-pyrolysis temperature, the post-pyrolysis temperature has a minor effect on the specific surface area. Post-pyrolysis lowers the surface area in general (Table S1†), but pre-pyrolysis temperature sets the range (Fig. 5), in terms of structural modifiability. The structure of carbon synthesized at lower pre-pyrolysis temperatures (*T*_1_ < 600 °C) is rather amorphous and therefore more modifiable. Therefore, lower pre-pyrolysis temperature allows for more burn-off at the later post-pyrolysis stage, which, in turn, allows for materials with lower surface areas.

The values of specific surface area calculated from Ar and N_2_ sorption data are comparable, indicating that the surface of synthesized carbons is free of functional groups, which would interact with N_2_.

### Electrochemical characterization

It has been proposed in several studies^42,44^ that the mechanism for storing sodium ions in hard carbon is a two-step process. It is believed that sodium ions first adsorb on the hard carbon surface at 0.2 V < *E* < 1.0 V *vs.* Na/Na^+^. Thereafter, the insertion of sodium ions into micropores that exist in the interlayer of local graphitic domains takes place (at 0 V < *E* < 0.2 V *vs.* Na/Na^+^). Therefore, the obtained hard carbon should have graphitic microcrystallites, but should not be fully graphitized. In this paper, our aim was to synthesize some hard carbon materials that would have the optimal graphitization degree *i.e.* a optimized surface area, specific interlayer distance, *I*_D_/*I*_G_ value and other properties characteristic of a hard carbon anode material.

Cycling performance and constant current (CC) discharge data (Fig. 7a–e) show very high capacity of 328 mA h g^−1^ with a plateau region (*E* < 0.2 V *vs.* Na/Na^+^) capacity of 243 mA h g^−1^ for PDC-450-1400 A at the current density of 50 mA g^−1^ (0.14C). At charging current of 25 mA g^−1^ (0.07C), even higher capacities were reached – 350 mA h g^−1^ with a plateau region capacity of 250 mA h g^−1^. The C-rates have been calculated for 350 mA h g^−1^ theoretical capacity (1C = 350 mA g^−1^). A long-term measurement demonstrates relatively high stability of PDC-450-1400 A. After about 130 charge/discharge cycles, the capacity stabilized at 280 mA h g^−1^, with the initial capacity of 320 mA h g^−1^ (Fig. 7e). PDC-450-1400 A exhibited a somewhat high ICE of 80% (Table 1 and Fig. S3†). The highest capacities were achieved with hard carbon materials that had low specific surface areas, broad 002 peak in XRD diffractograms, interlayer spacing of about 3.9 Å and optimal *I*_D_/*I*_G_ values around 1.50 (PDC-450-1400 A for example). This might indicate the existence of microcrystallites and provide an explanation for the high capacities.

**Table 1:** Electrochemical performance metrics of studied hard carbon materials

Sample	ICE	*Q*_0.07C_/*E* = 1.5V (mA h g^−1^)	*Q*_0.07C_/*E* < 0.2V (mA h g^−1^)	*Q*_0.14C_/*E* = 1.5V (mA h g^−1^)	*Q*_0.14C_/*E* < 0.2V (mA h g^−1^)	*Q*_0.3C_/*E* = 1.5V (mA h g^−1^)	*Q*_0.3C_/*E* < 0.2V (mA h g^−1^)
PDC-300-1400 A	77%	321	233	256	178	161	87
PDC-450-1000 A	74%	301	174	278	166	262	154
PDC-450-1100 A	75%	328	205	294	189	268	167
PDC-450-1200 A	75%	326	217	301	204	277	186
PDC-450-1300 A	78%	329	233	295	210	242	161
**PDC-450-1400 A**	**80%**	**356**	**262**	**332**	**245**	**300**	**221**
PDC-450-1500 A	74%	333	246	257	194	163	103
PDC-500-1000 A	62%	250	133	225	127	206	111
PDC-500-1100 A	64%	248	144	212	124	191	106
PDC-500-1200 A	71%	300	201	252	171	240	162
PDC-500-1300 A	70%	224	162	163	117	168	115
PDC-500-1400 A	73%	306	233	201	140	164	102
PDC-600-1000 A	39%	94	43	73	29	60	20
PDC-600-1100 A	37%	84	43	60	25	47	14
PDC-600-1200 A	51%	142	99	73	39	50	18
PDC-600-1300 A	49%	134	95	70	37	46	16
PDC-600-1400 A	58%	216	165	131	92	59	24
PDC-700-1000 A	50%	147	69	127	57	103	41
PDC-700-1100 A	51%	158	83	137	75	113	54
PDC-700-1200 A	56%	206	140	121	70	73	24
PDC-700-1300 A	66%	279	209	197	138	112	57
PDC-700-1400 A	67%	290	223	180	125	113	63
PDC-800-1000 A	33%	128	53	109	50	98	42
PDC-800-1300 A	43%	165	106	123	73	93	48
PDC-800-1400 A	50%	188	135	142	94	101	56

### Effect of synthesis steps on capacity

**Fig. 6:**
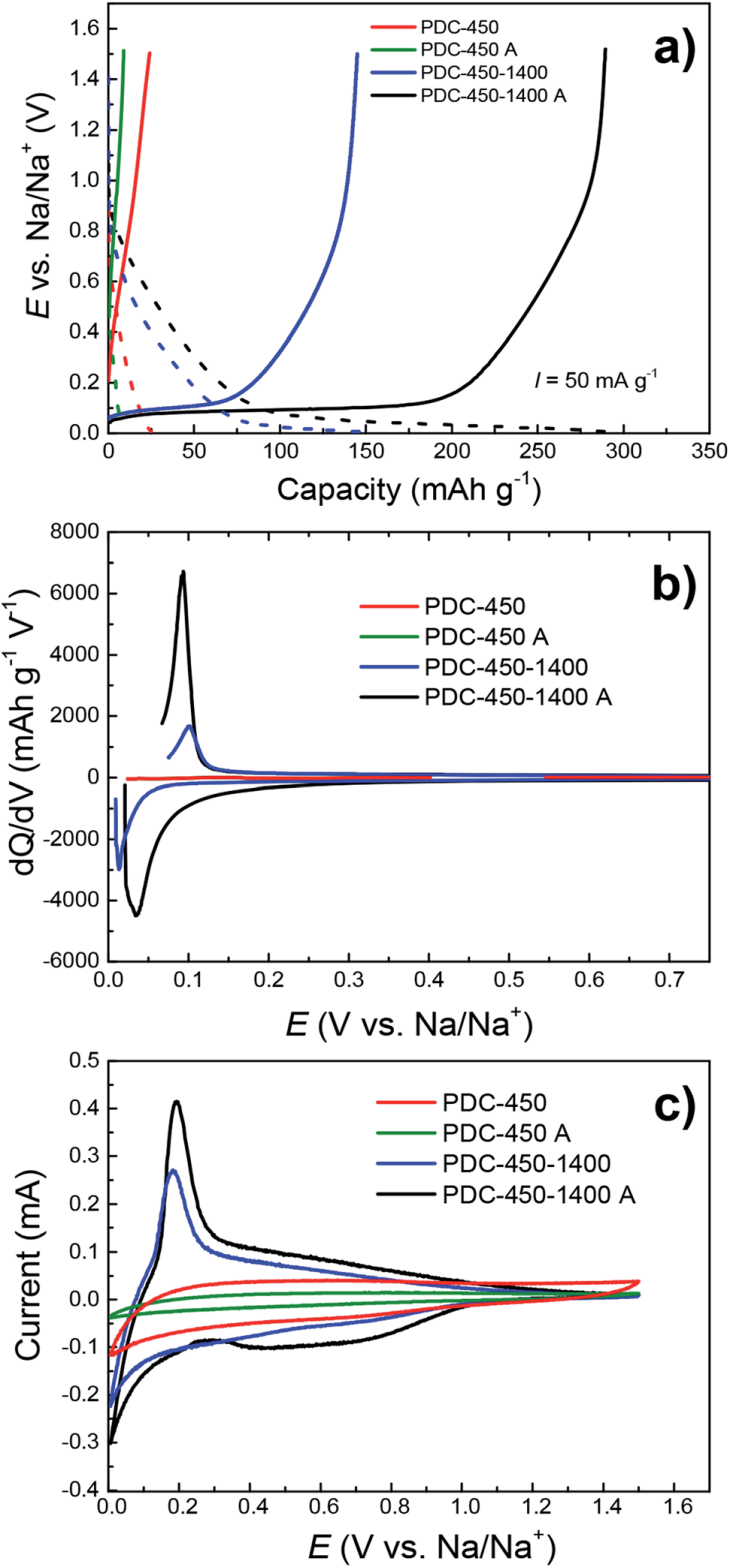
CC charge/discharge (a), d*Q*/d*V* (b) and CV (c) curves with the potential sweep rate of 0.1 mV s^−1^, demonstrating the effect of synthesis steps (A appended if KOH–HCl treatment was used) on capacity in NaPF_6_ EC : PC (1 : 1) electrolyte.

The need for all three synthesis steps (pre-pyrolysis, KOH and HCl treatment, post-pyrolysis) is demonstrated in Fig. 6.

PDC-450-1400 A–the hard carbon treated with all 3 synthesis steps has the highest capacity, while the materials synthesized with fewer steps have significantly lower capacities. The lower capacities of PDC-450 and PDC-450-1400 can be explained by differences in their elemental composition, since these hard carbons have very high impurity content (Table S2†), which cause the graphitization of pre-pyrolysed materials. As mentioned before, it has been demonstrated in previous research papers^40,45^ that elements like Fe and Ca can act as catalysts for the graphitization of carbon structure. Therefore we suggest, that the lower capacities of PDC-450 and PDC-450-1400 are caused by higher degree of graphitization, which in turn, is caused by differences in materials' elemental composition.

Surprisingly, when comparing PDC-450 and PDC-450 A, the latter has a somewhat lower capacity of the two. Since both carbons demonstrated very low capacities, the difference was also small, only 16 mA h g^−1^ with no identifiable plateau region. This is due to KOH and HCl treatment steps, during which the material might have become porous to an extent that is disruptive for sodium storage. A similar phenomenon was noted by Dou *et al.*,^30^ where the carbonaceous material was activated with phosphoric acid.

### Effect of pre-pyrolysis temperature on capacity

**Fig. 7:**
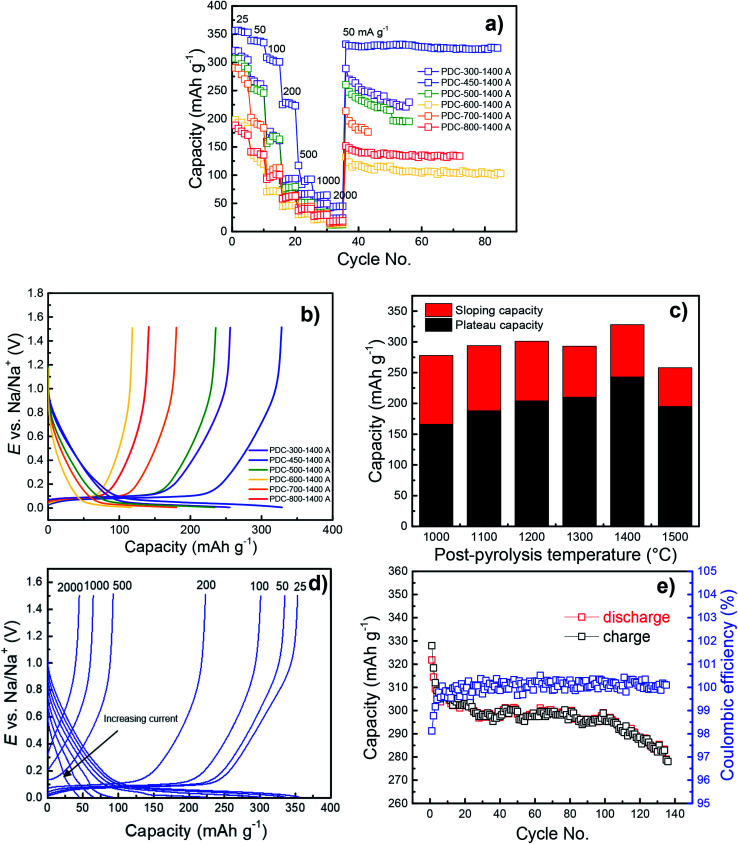
Cycling performance (a) and CC charge/discharge curves at 50 mA g^−1^ (b) for PDC-*T*_1_-1400 A. Plateau and sloping capacities for PDC-450-*T*_2_ A at 50 mA g^−1^ (c), CC charge/discharge curves for PDC-450-1400 A at different current densities (25–2000 mA g^−1^) (d) and a lifetime measurement for PDC-450-1400 A at 25 mA g^−1^ (e).

The pre-pyrolysis temperature is one of the key factors, which influences the performance of the peat-derived hard carbon in the half-cell charge–discharge measurements. Fig. 7a–e demonstrate that regardless of post-pyrolysis temperature, lower pre-pyrolysis temperatures allow for higher capacities, increasing from 141 mA h g^−1^ to 328 mA h g^−1^, the capacities of PDC-800-1400 A and PDC-450-1400 A, respectively. In part, this is due to lower specific surface areas of the materials prepared at lower *T*_1_ (Fig. 5), causing higher ICE values (Fig. S3†), which in turn causes higher capacities. Also, hard carbons with higher pre-pyrolysis temperatures (*T*_1_ > 500 °C) have higher impurity content, which lowers their electrochemical capacities.

Although PDC-300-1400 A also has a relatively high capacity, its capacity does not surpass that of PDC-450-1400 A (Fig. 7b). Regarding the TGA data, PDC-300 has still a lot of mass to be burned off at higher temperatures. This implies that at 300 °C, there may be different organic compounds in the material or structural properties that lower the capacity.

For a hard carbon to store sodium, it needs to have small graphitic domains^19^ and when pyrolyzing peat at 300 °C, it might be too amorphous to allow for effective sodium intercalation (Fig. 4a). The CC measurements data for PDC-600-1400 A also demonstrate much lower capacity that could be expected from the otherwise stable trend (the higher the pre-pyrolysis temperature, the lower capacity). Reasons for this can be found from the carbon characterization results.

As discussed earlier, the hard carbons obtained at 600 °C have significantly higher impurity content, higher surface area, smaller particles and a higher degree of graphitization. This suggests the formation of smaller particles in the pre-pyrolysis step that are more thoroughly graphitized, have less amorphous fraction and are therefore less efficient in sodium storage. In conclusion, lower pre-pyrolysis temperatures lead to materials with higher capacity, but the trend is not definite and may depend on the biomass precursor used.

### Effect of post-pyrolysis temperature on capacity

The post-pyrolysis temperature has little effect on the capacity compared to pre-pyrolysis temperature. As a general trend, higher post-pyrolysis temperature leads to higher capacity (Fig. S4†), but the biggest impact the higher post-pyrolysis temperature has, is on the plateau capacity (Fig. 7c). Plateau capacities increase significantly (from 166 to 245 mA h g^−1^) with the increase of post-pyrolysis temperature, which is probably caused by slight structural disordering in the carbon structure.

However, Fig. 7b and c demonstrate that PDC-450-1500 A deviates from this trend, as both the sloping and plateau capacity decrease significantly (257 mA h g^−1^ and 194 mA h g^−1^, respectively). A similar effect has been noted in the literature.^29^

The increase of the plateau capacity is probably caused by burn-off of amorphous carbon, which makes the local graphitic domains more attainable and prominent and therefore allows for better sodium storage capability. Post-pyrolysing of the carbonaceous materials also lowers the surface area, which also leads to higher ICE values (Table 1).

### The influence of electrolyte

The measurements of the materials prepared in this work were carried out in 1 M NaPF_6_ EC : PC (1 : 1) as well as 1 M NaClO_4_ EC : DEC (1 : 1) electrolytes in order to compare two of the most common electrolyte solutions and their effect on the electrochemical behaviour of the carbons obtained. Fig. S4† demonstrates that better results have been established for the 1 M NaClO_4_ EC : DEC (1 : 1) based system. Both the capacity at 1.5 V and plateau capacity are remarkably higher in the case of PDC-450-(1000-1500) A based half-cells, where the plateau capacity increases from 218 to 245 mA h g^−1^. This might be partly due to the significantly lower dielectric constant of the EC : DEC mixture. EC has a dielectric constant of 89.78, whereas for DEC it's 2.81 (ref. 46) and therefore the migration of sodium ions might have been enhanced. The potential co-intercalation of PC^47^ and the possible impurities in NaPF_6_ might also have been the reasons for this discrepancy in the measurements.

## Conclusions

A complex synthesis method has been developed for the preparation of the hard carbon materials from highly decomposed Estonian peat. Hard carbons were obtained by pre-pyrolysing, followed by treating with base and acid solutions and finally by post-pyrolysing the material. We have synthesized some hard carbon materials that demonstrated high capacities in sodium-ion battery half-cells and according to physical characterizations data, the optimal graphitization degree and level of purity has been achieved to allow for effective sodium storage. The combination of lowering the pre-pyrolysis temperature and increasing the post-pyrolysis temperature has an enormous effect on the structural properties of the hard carbons while maintaining some of the peat's cellular structure. Using the multiple synthesis steps enables to change the ordering of the carbon structure and impact the surface area – carbon materials with surface areas as low as 6 m^2^ g^−1^ were prepared. Peat contains many impurities (mostly Ca and Fe hydroxides), which lower the capacity significantly. However, the KOH and HCl treatment steps are extremely effective in removing these impurities from materials that have been previously pyrolyzed at lower temperatures (300–500 °C), enhancing noticeably the half-cell capacity. However, the removal of impurities is not effective in the case of the materials pre-pyrolysed at higher temperatures (600–800 °C). These materials become too graphitic in the post-pyrolysis stage and therefore have very low capacities (of ∼130 mA h g^−1^). However, the KOH and HCl treatment steps of the hard carbons pre-pyrolysed at 450 °C, elevate the capacity from 141 to 328 mA h g^−1^. The best results have been obtained for PDC-450-1400 A – the peat material pre-pyrolysed at 450 °C, treated with KOH and HCl and post-pyrolysed at 1400 °C. PDC-450-1400 A demonstrated great sodium storage capability, achieving a reversible capacity of 330 mA h g^−1^ with a plateau region of 245 mA h g^−1^ at 50 mA g^−1^ current density (350 mA h g^−1^ with a plateau of 250 mA h g^−1^ at 25 mA g^−1^) and an 80% initial coulombic efficiency.

Based on the analysis of the electrochemical results the half-cells completed it has been established that peat can successfully be used as a precursor for obtaining the high capacity hard carbon electrode materials for the sodium-ion batteries.

## Author contributions

A. Adamson and R.Väli conceived the idea. A. Adamson and M. Paalo synthesised the hard carbon materials. A. Adamson performed the electrochemical measurements and analysed the data. J. Aruväli performed the XRF and XRD measurements and analysed the data. M. Koppel and R. Palm conducted the gas sorption measurements and analysed the data. E. Härk performed the TEM measurements. J. Nerut performed the TGA measurements and analysed the data. T. Romann performed the Raman measurements and A. Adamson analysed the data. E. Lust and A. Jänes provided the funding for the research paper and contributed to the idea. A. Adamson wrote the manuscript. R. Väli, E. Lust and A. Jänes edited the manuscript and supervised. All authors have given approval to the final version of the manuscript.

## Conflicts of interest

There are no conflicts to declare.

## Supplementary Material

RA-010-D0RA03212C-s001
