# Supplementary material for: Peat-derived hard carbon electrodes with superior capacity for sodium-ion batteries
Source: RSC Adv. 2020 May 27;10(34):20145–54. doi: 10.1039/d0ra03212c (PMC9054124; doi:10.1039/d0ra03212c)
Supplement: RA-010-D0RA03212C-s001 [file RA-010-D0RA03212C-s001.pdf]

Electronic Supplementary Material (ESI) for RSC Advances.
This journal is © The Royal Society of Chemistry 2020

Supplementary material

Peat-derived hard carbon electrodes for superior capacity in sodium-ion batteries

A. Adamson[1], R. Väli[1], M. Paalo[1], J. Aruväli[2], M. Koppel[1], R. Palm[1], E. Härk[3], J. Nerut[1], T. Romann[1], E. Lust[1], A. Jänes[*,1]

[1]Institute of Chemistry, University of Tartu, Ravila 14a, 50411, Tartu, Estonia

[2]Institute of Ecology and Earth Sciences, University of Tartu, Ravila 14a, 50411, Tartu, Estonia

[3]Institute Electrochemical Energy Storage (EM-IEES), Helmholtz-Zentrum für Materialien und Energie GmbH, Hahn-Meitner-Platz 1, 14109 Berlin, Germany

[*]Corresponding author. Phone: +372 737 5178; fax: +372 737 5264

E-mail addresses: alar.janes@ut.ee

Table S1. EDX results of analyzed carbon samples.

Carbon sample	C mass %	O mass %	N mass %	Si mass %	S mass %	Ca mass %
PDC-450 A	76.78	17.04	5.49	0.38	0.31	0
PDC-450-1000 A	96.40	2.77	0	0.30	0.30	0.31
PDC-450-1200 A	96.96	1.72	0	0.61	0.25	0.46
PDC-450-1400 A	98.15	1.24	0	0.36	0	0.36

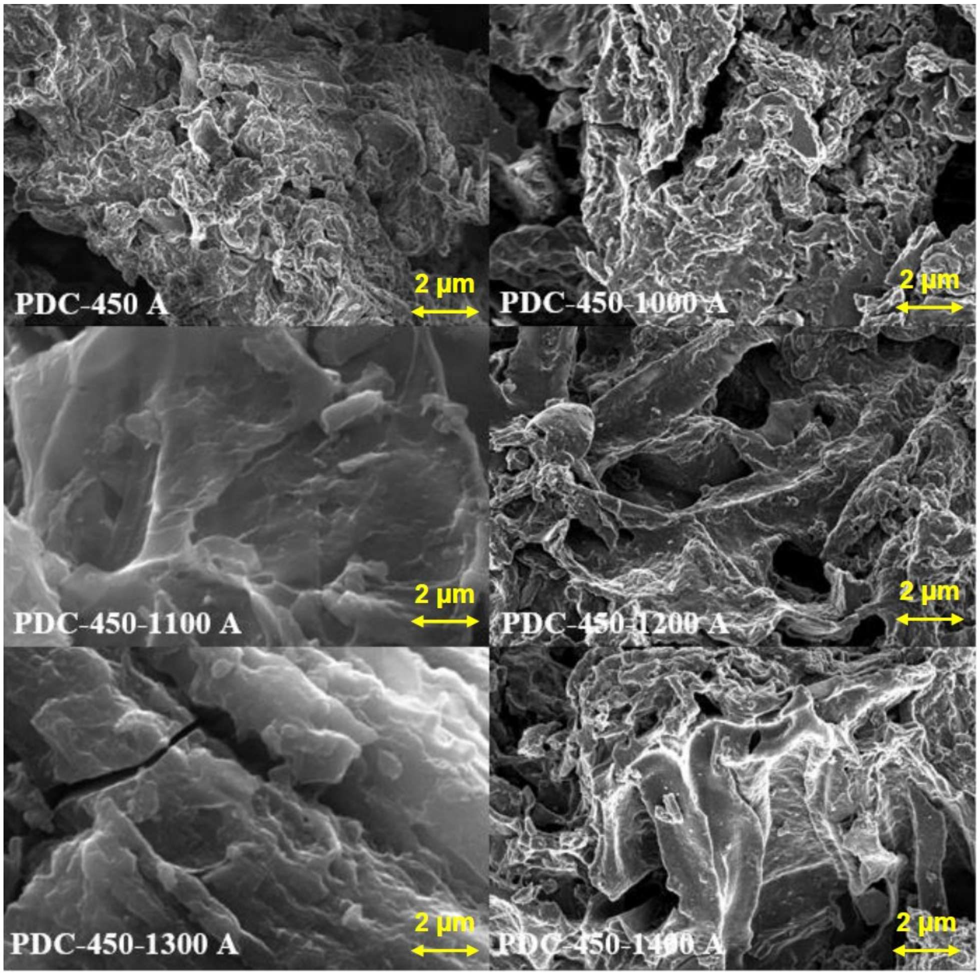

Figure S1. SEM images of PDC-450 A and PDC-450-(1000-1400) A prepared at different post-pyrolysis temperature from 1000 to 1400 °C, noted in figures.

Table S2. Properties of obtained hard carbons.

Sample	Wt%	Specific surface area ($m^2\ g^{-1}$)	Ca%	Fe%	Cl%	I_D/I_G	$FWHM_G$	Particle size (μm)
PDC-300	59.9%							
PDC-300 A	72.9%							
PDC-300-1400 A	52.8%					1.119	82.735	
PDC-450	32.4%	13	19.3	0.4	0	0.611	93.335	5.99
PDC-450 A	77.9%	5	0.38	0.7	0			
PDC-450-1400								
PDC-450-800 A	72.9%	334				0.708	70.211	
PDC-450-1000 A	71.6%	210				0.555	81.473	
PDC-450-1100 A	71.2%	40				0.887	74.084	
PDC-450-1200 A	69.3%	9				0.970	72.023	
PDC-450-1300 A	68.3%	7				1.063	70.812	
PDC-450-1400 A	67.5%	6				1.154	69.594	
PDC-450-1500 A	64.9%	7				1.452	66.938	
PDC-500	24.8%	35	13.4	4.7	0	0.518	94.723	5.83
PDC-500 A	61.8%	104						
PDC-500-1000 A	64.8%	363				0.791	71.871	
PDC-500-1100 A	64.6%	309				0.934	66.269	
PDC-500-1200 A	65.1%	29				1.056	71.284	
PDC-500-1300 A	59.5%	25				1.070	67.777	
PDC-500-1400 A	60.5%	19				1.266	67.844	
PDC-600	35.8%	65	10.2	3.6	0.1	0.442	69.216	3.79
PDC-600 A	93.2%	155	5.5	2	5.6			
PDC-600-1000 A	82.1%	270				1.070	71.949	
PDC-600-1100 A	75.5%	266				1.006	72.681	
PDC-600-1200 A	75.1%	91				1.077	71.140	
PDC-600-1300 A	71.4%	98				1.203	69.817	
PDC-600-1400 A	68.2%	101				1.140	71.500	
PDC-700	32.3%	230						6.67
PDC-700 A	87.7%	277						
PDC-700-1000 A	90.3%	348				1.044	68.201	
PDC-700-1100 A	83.5%	402				0.961	70.126	
PDC-700-1200 A	79.1%	83				1.122	69.563	
PDC-700-1300 A	75.0%	78				1.312	66.914	
PDC-700-1400 A	73.9%	87				1.412	66.338	
PDC-800	34.7%	270	8.2	2.6	0.1			6.86
PDC-800 A	78.3%	357	2.7	2.6	4.8			
PDC-800-1000 A	99.8%	314				1.076	70.655	
PDC-800-1300 A	99.7%	290				1.044	64.911	
PDC-800-1400 A	99.7%	222				1.185	68.390	

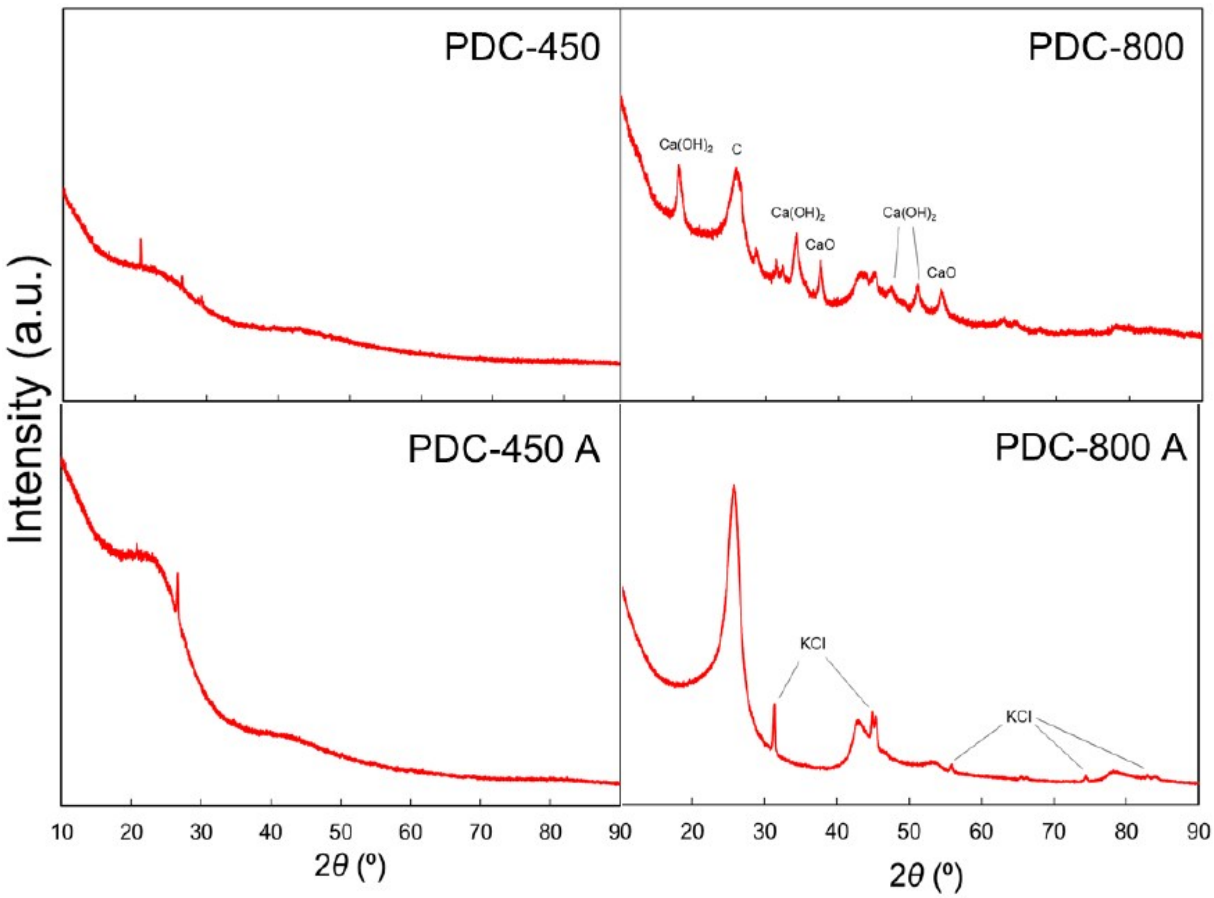

Figure S2. Diffractograms displaying the impurities in PDC-800 and PDC-800 A.

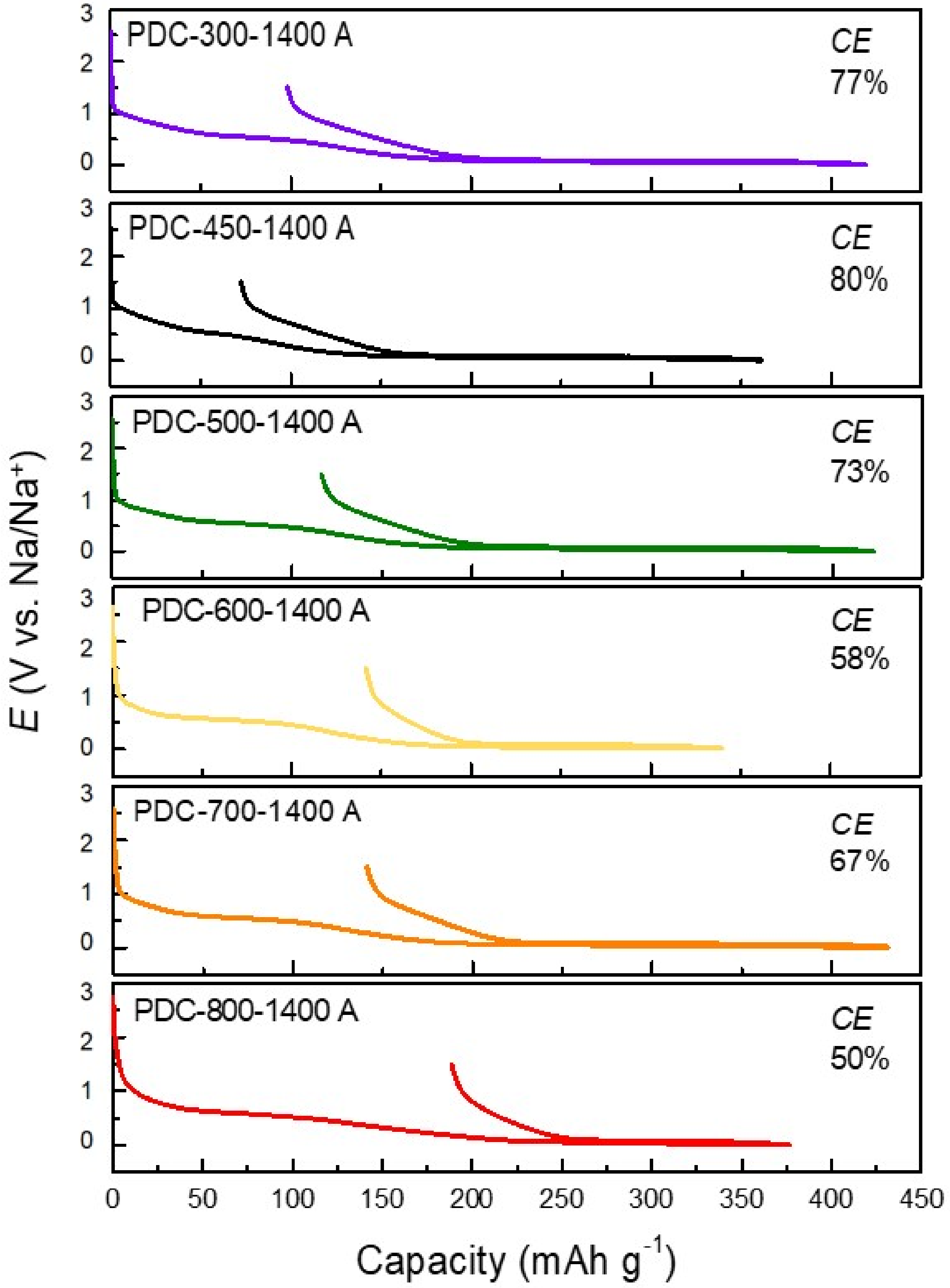

Figure S3. Galvanostatic profiles of the first cycle for PDC-T_1-1400 A hard carbons with coulombic efficiency values.

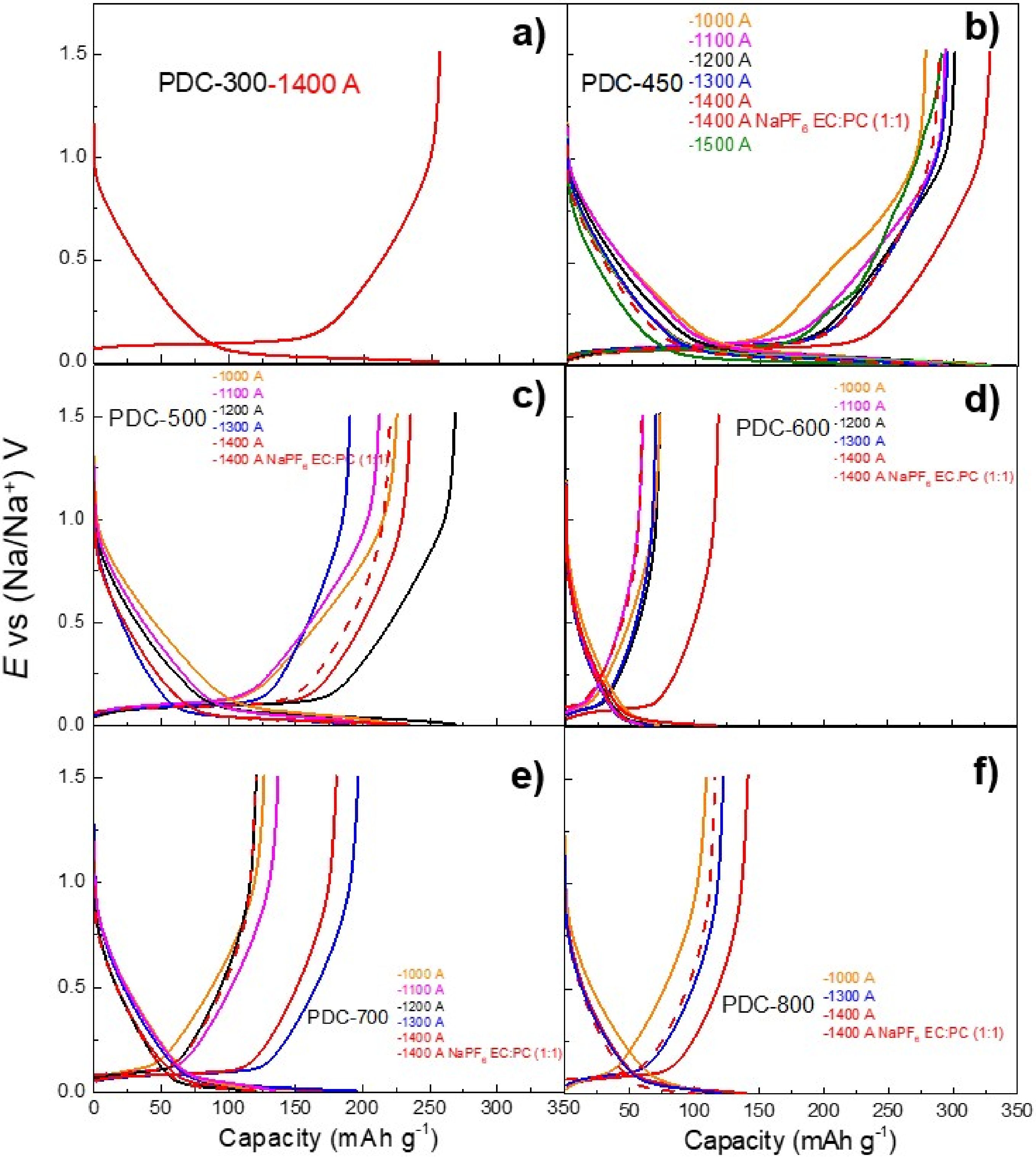

Figure S4. Discharge curves for $NaClO_4$ + EC:DEC (1:1) electrolyte based half-cells at 50 mA g^{-1} current density. For comparison, the data for selected carbon in 1 M $NaPF_6$ + EC:PC (1:1) are given.
